# Follow-up Routines Matter for Adherence to Endocrine Therapy in the Adjuvant Setting of Breast Cancer

**DOI:** 10.1177/11782234241240171

**Published:** 2024-04-15

**Authors:** Carolina Aurell, Alaa Haidar, Daniel Giglio

**Affiliations:** 1 Department of Surgery, Halland Hospital Varberg, Region Halland, Varberg, Sweden; 2 Department of Oncology, Institute of Clinical Sciences, Sahlgrenska Academy, University of Gothenburg, Gothenburg, Sweden; 3 Department of Oncology, Halland Hospital Halmstad, Region Halland, Halmstad, Sweden; 4 Department of Oncology, Sahlgrenska University Hospital, Gothenburg, Sweden

**Keywords:** Breast cancer, endocrine therapy, aromatase inhibitor, tamoxifen, adherence

## Abstract

**Background:**

Endocrine therapy (ET) adherence leads to increased survival in breast cancer (BC). How follow-up should be done to maximize adherence is not known.

**Objectives:**

To assess adherence to ET, factors favouring adherence to ET and effects on survival in a population-based cohort of BC patients in western Sweden.

**Design:**

This is a retrospective study.

**Methods:**

We included 358 patients operated for oestrogen receptor-positive BC and recommended 5 years of ET, in Region Halland, Sweden, year 2015 to 2016. Demographical, clinical and pathological data and use of ET were retrieved from the electronic medical records. Patients were considered adherent if taking ET for 5 years or during the full extent of the follow-up, until termination of ET due to BC relapse or death and where renewals of prescriptions of ET covered ⩾80% of the ordinated dose. Two follow-up routines were employed, ie, routine A where patients were contacted annually by nurses and a more passive follow-up routine B where patients were only contacted by nurses at 2 years and 5 years following start of ET.

**Results:**

Medication persistence for 4 years and more was good and similar between patients initiating aromatase inhibitor (AI) and tamoxifen (75.7% and 72.0%, respectively, *P* = .43). More patients initiating AIs changed ET due to side effects compared with patients initiating tamoxifen (24.3% vs 9.9%, respectively, *P* < .0001). Endocrine therapy adherence was better for follow-up routine B than for follow-up routine A (hazard ratio [HR] = 2.71 [1.44-5.09], *P* = .0027).

**Conclusions:**

Adherence to ET in BC is high in Western Sweden. Less regular nurse-initiated contacts between BC patients and nursesled surprisingly to a better adherence than a more regular nurse-initiated contact.

## Plain Language Summary

Follow-up routines are important for adherence to anti-hormonal therapy after breast cancer surgery

In this study conducted in western Sweden, researchers looked at how well breast cancer (BC) patients followed their prescribed endocrine therapy (ET) for 5 years, which is crucial for their survival. They studied 358 patients diagnosed with oestrogen receptor-positive BC between 2015 and 2016. The study compared two follow-up routines: one where patients were contacted annually by nurses (routine A) and another where patients were contacted only at 2 years and 5 years after starting ET (routine B). Surprisingly, patients in routine B, with less frequent nurse contacts, were more likely to adhere with their ET compared with those in routine A. The study also found that patients taking aromatase inhibitors (AIs) were more likely to switch their ET due to side effects compared with those taking tamoxifen, but overall, adherence rates were similar between the 2 groups. In summary, the study showed that BC patients in western Sweden generally followed their prescribed ET well. In addition, having less frequent nurse-initiated contacts surprisingly improved patient adherence with their treatment.

## Introduction

Breast cancer (BC) is the most common cancer form among women where BC positive for the estrogen receptor (ER) accounts for over 80% of all BC.^1-4^ Adjuvant treatment after surgery with 5 year endocrine therapy (ET) in the form of tamoxifen or aromatase inhibitors (AIs) contributes significantly to increased survival in ER-positive BC.^5,6^ Endocrine therapy is, however, associated with side effects such as hot flushes, night sweats, oedema and vaginal dryness, which may lead to poor compliance and early discontinuation.^7^ Other factors associated with poor compliance are comorbidity, young age and high out-of-pocket costs for ET.^8-10^ Poor compliance may consequently lead to increased BC recurrence and worse overall survival (OS).^11,12^ Studies show that BC recurrence continues to occur after 5 years after diagnosis and ET for 10 years leads to increased BC-specific survival compared with ET for 5 years.^13-15^ There is therefore an essential need to identify follow-up routines to support BC women on ET and to enable compliance to ET and prevent discontinuation. How follow-up should be conducted to optimize compliance is at present not known. Studies show that regular contact with a nurse leads to better compliance to ET in BC patients.^16,17^ Trials are also ongoing to assess the efficacy of telephone-based training programmes to cope with side effects of ET, text message reminders and telephone-based counselling to improve adherence to ET in BC patients.^18,19^

Surgery and adjuvant therapy against BC are given at Varberg Hospital and Halmstad Hospital in Region Halland in western Sweden. The centres have employed different follow-up routines for patients on ET due to BC, ie, the contact with nurses has either been more regular and more initiated by nurses or less regular and more initiated by the patients. Here, we assessed whether adherence to ET in BC patients is affected by the degree of nurse-driven contact with the patients. Moreover, we assessed which factors that correlate with adherence to ET and how changes of ET occur over time in the adjuvant setting of BC.

## Methods

**Figure 1.**
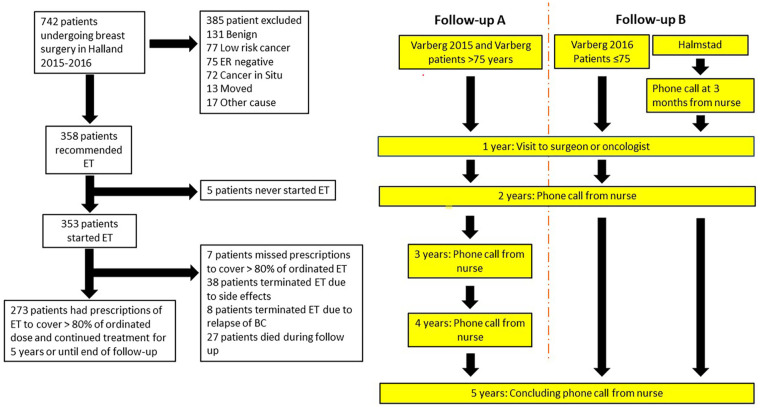
Flow chart of breast cancer patient treatments, adherence to endocrine therapy and follow-up routines.

All patients undergoing surgery for breast lumps in 2015 to 2016 in Region Halland, Sweden, were identified in the electronic medical records. The cohort consisted of 743 patients where 385 patients were excluded from the study as they did not meet inclusion criteria (Figure 1). The study cohort consisted of 358 patients who met inclusion criteria, ie, they were diagnosed and operated for ER+ (⩾10%)-positive BC and were recommended adjuvant ET at a multidisciplinary conference board where surgeons, oncologists, pathologists and radiologists attended. All patients were informed about their tumour type and lymph node status. Moreover, all patients included were traceable during the follow-up time, ie, staying in the region during the follow-up time. Demographical data, clinical data and pathological data were retrieved from the electronic medical records and registered in a database. The initial recommended ET, any change of ET either due to side effects, the patient’s own choice or due to predetermined switch to other ET, causes of change of ET, side effects of ET and points in time for change of ET were registered in the database. Moreover, the frequency of renewals of prescriptions of ET was recorded. Any relapse of BC and death during the follow-up time were registered in the database. Cut-off date was set on December 17, 2020.

All patients were prescribed at least 5 years of ET. The patient was considered adherent to the initial ET plan if the patient took ET during 5 years or during the full extent of the follow-up time, until termination of ET due to BC relapse or death and if renewals of prescriptions of ET covered at least 80% of the ordinated dose during the follow-up time. Patients were also considered non-adherent if they decided to stop ET after discussion with the oncologist or surgeon. Patients were followed until day of pre-termination of ET or when day of non-adherence occurred, death, BC relapse or cut-off date. Information on cause of death was retrieved from the medical chart of the patient. Whether the cause of death was BC, other cause or if information on cause of death was missing was also retrieved from the medical chart. Follow-up routines differed between Varberg Hospital and Halmstad Hospital due to different follow-up routines developed over time at the two hospitals. At both hospitals the patient had a follow-up meeting with a surgeon or an oncologist at 1 year, a phone call from a nurse at 2 years and a concluding phone call from a nurse at 5 years following start of ET. At Halmstad Hospital, a follow-up phone call from a nurse was made 3 months following initiation of ET. At Varberg Hospital, a more active nurse-driven follow-up routine (follow-up A) was employed in patients diagnosed in 2015 and before, ie, a phone call from a nurse was made here also at 3 years and at 4 years after initiation of ET (see Figure 1). Patients were informed to contact the clinic for renewal of prescription of ET, if intolerable side effects to ET appeared and/or wanting to make any change in ordinated ET. In the analysis, follow-up routines were subdivided into a more active nurse-driven follow-up routine A (119 patients, 33.7%) employed at Varberg Hospital in 2015 and a more patient-driven follow-up routine B (234 patients, 60.3%) employed at Halmstad Hospital and at Varberg Hospital in 2016 (see Figure 1). As noted in Figure 1, patients who became 75 years old during follow-up time or older were followed according to routine A in Varberg, regardless of which year the patient had BC surgery.

### Statistics

No power analysis was performed since we included all patients operated for BC during 2015 to 2016 in Region Halland. The χ^2^-test was used to compare categorical variables between groups. The Mann-Whitney test was used to compare continuous variables. Bivariate logistic regression analysis was performed and crude odds ratios and adjusted odds ratios were calculated for explanatory variables correlating with the dichotomous dependent variable adherence (adherent vs non-adherent). Adherence to ET over time was depicted with the Kaplan-Meier analysis and adherence between groups was compared using a 2-sided log-rank test (Mantel-Cox). Cases where termination of ET was due to progressive disease or death were censored. Statistical significance was set at *P* values less than .05. Mean value ± standard error of the mean (SEM) is given in the text or median (95% confidence interval) where indicated. IBM SPSS Statistics version 26 (IBM Corp, Armonk, NY, USA) and GraphPad Prism program 9.1.0 (GraphPad Software, Inc, San Diego, CA, USA) were used to analyse data.

## Results

### Description of the cohort

Demographical, clinical and pathological data are displayed in Table 1. Median age in the cohort was 65.5 years. In the cohort, 6.8% of patients were previously diagnosed with ipsilateral breast. Patients with bilateral and multifocal BC constituted 1.7% and 9.9% of the cohort, respectively. Non-special type carcinoma (NST) and lobular carcinoma constituted 76.5% and 15.9%, respectively, of the breast tumours. T1 tumours were most common (58.1%) and 27.9% of the cohort had lymph node metastases. Breast tumours were positive for HER2 in 11.9% of cases and trastuzumab was given to 76.2% of patients with HER2-positive BC. Adjuvant radiotherapy and chemotherapy were given to 72.2% and 32.3% of the cohort, respectively. During follow-up, 9.1% of patients had recurrence (local + distant) and 1.1% of the cohort were diagnosed with a contralateral BC. During follow-up, 8.8% died and 2.5% died of BC.

**Table 1.** Characteristics of patients, surgical and oncological treatments and tumour characteristics.

	Number of patients	%
**Hospital**		
Halmstad	173	49.0
Varberg	180	51.0
**Age groups**		
31-40	6	1.7
41-50	39	11.0
51-60	76	21.5
61-70	120	34.0
71-80	84	23.8
81-90	28	7.9
**Gender**		
Female	352	99.7
Male	1	0.3
**HRT at diagnosis** ^a^		
No	120	70.2
Yes-systemic	28	16.4
Yes-local	23	13.5
**Breast surgery**		
Lumpectomy	197	55.8
Mastectomy	156	44.4
**Lymph node dissection**		
Yes	97	27.5
No	256	72.5
**Chemotherapy**		
Yes	114	32.3
No	239	67.7
**Radiotherapy**		
Yes	255	72.2
No	98	27.8
**Trastuzumab treatment**		
Yes	32	9.1
No	321	90.9
**Type**		
Carcinoma of no special type	270	76.5
Lobular carcinoma	56	15.9
Mixed	7	2.0
Other type	19	5.4
No data	1	0.3
**Tumour size**		
T1 (⩽20 mm)	205	58.1
T2 (21-50 mm)	120	34.0
T3 (>50 mm)	21	6.0
T4	5	1.4
No data	2	0.6
**Lymph nodes** ^b^		
N0	231	68.3
N submicro/micro	13	3.8
N1	71	21.0
N2	20	6.0
N3	3	0.9
**Stage**		
I	147	41.6
II	156	44.2
III	33	9.3
Unknown (stage ⩾ I < IV)	17	4.8
**Grade**		
Grade 1	61	17.3
Grade 2	235	66.6
Grade 3	53	15.0
No data	4	1.1
**HER2**		
Negative	311	88.1
Positive	42	11.9

a In 173 patients (45.6% of the cohort), there was no information on hormone replacement therapy in the medical records.

b Not included are patients not lymph node operated or where sentinel node was not found.

### Adherence to endocrine therapy

**Figure 2.**
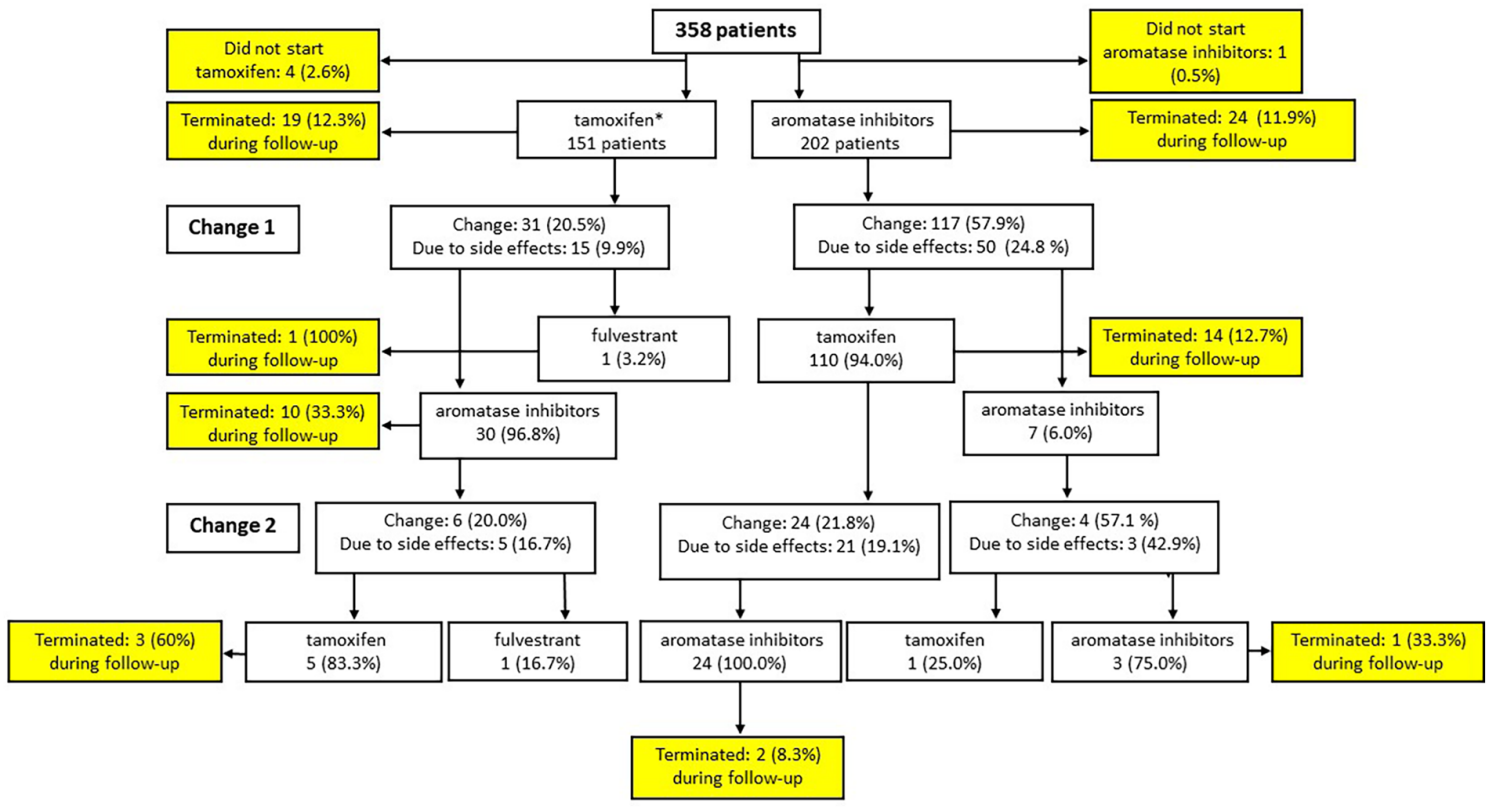
Flow chart of changes and termination of ET in breast cancer patients.

Among patients offered ET, 43.3% were prescribed tamoxifen and 56.7% AIs (letrozole, anastrozole, exemestane). Five patients were ordinated ET but did not initiate ET (1.3%). Median time from start of ET to end of follow-up was 4.66 (4.44-4.91) years and 4.49 (4.35-4.61) years for follow-up A and follow-up B, respectively (*P* = .90). Planned switch from AI to tamoxifen was more common than from tamoxifen to AI, ie, 34.7% (70/202) and 4.0% (6/151) of cases, respectively (*P* < .0001). Among patients initiating AI, 24.8% changed to tamoxifen or other AI due to side effects, whereas 9.9% of patients initiating tamoxifen changed to AI or fulvestrant due to side effects (*P* = .0004; Figure 2).

**Figure 3.**
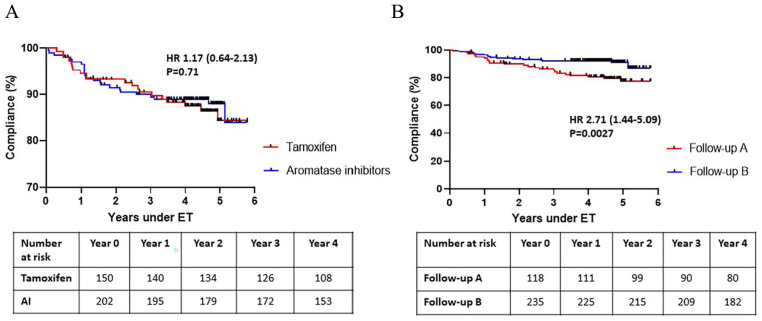
Adherence to endocrine therapy depending on first choice of endocrine therapy (A) and follow-up (B).

In average, patients prescribed tamoxifen had their first change of ET after 57 ± 13 weeks compared with after 61 ± 7 weeks in patients prescribed AI (*P* = .82). No difference in median time on ET was observed in patients who initiated treatment with AI compared with patients initiating treatment with tamoxifen (55.2 [53.1-56.8] vs 53.7 [51.6-55.3] months, respectively, *P* = .07). Adherence of tamoxifen and AI was similar (hazard ratio [HR] = 1.17 [0.64-2.13]; *P* = .71; Figure 3A) and medication persistence for 4 years and more was 72.0% for patients initiating tamoxifen and 75.7% for patients initiating AI (*P* = .43; Table 2). Adherence to ET was better for the follow-up routine B than for the follow-up routine A (HR = 2.71 [1.44-5.09], *P* = .0027; Figure 3B and Table 2) and follow-up routine A constituted an independent factor associated with worse adherence (adjusted odds ratio [AOR]: 4.48 [1.33-15.02], *P* = .02; Table 3). Experiencing ET side effects was also an independent factor correlating with worse adherence to ET (AOR = 3.13 [1.30-7.50]).

**Table 2.** Number of years on endocrine therapy.

	<1 year n (%)	1 < 2 years n (%)	2 < 3 years n (%)	3 < 4 years n (%)	>4 years n (%)
Tamoxifen	10 (6.7)	6 (4.0)	8 (5.3)	18 (12.0)	108 (72.0)
AI	7 (3.5)	16 (7.9)	7 (3.5)	19 (9.4)	153 (75.7)
Follow-up A	7 (5.9)	12 (10.2)	9 (7.6)	10 (8.5)	80 (67.8)
Follow-up B	10 (4.3)	10 (4.3)	6 (2.6)	27 (11.5)	182 (77.4)

**Table 3.** Bivariate logistic regression analysis on explanatory variables correlating with adherence to endocrine therapy.

	COR (95% CI)	Significance	AOR (95% CI)	Significance
**Hospital**		*P* = .11		*P* = .39
Halmstad	Reference		Reference	
Varberg	1.73 (0.90-3.34)		0.56 (0.15-2.09)	
**Age groups**		*P* = .43		*P* = .69
⩽65 years	Reference		Reference	
>65 years	0.77 (0.41-1.47)		1.17 (0.53-2.59)	
**HRT at diagnosis**		*P* **=** **.16**		*P* = .10
No	Reference		Reference	
Yes-systemic	2.52 (0.91-7.01)	*P* = .08	2.16 (0.68-6.90)	*P* = .19
Yes-local	0.34 (0.04-2.75)	*P* = .31	0.24 (0.03-2.18)	*P* = .21
No data	0.99 (0.48-2.03)	*P* = .97	0.62 (0.27-1.42)	*P* = .25
**Radiotherapy**		*P* = .45		*P* = .74
No	Reference		Reference	
Yes	1.30 (0.65-2.58)		1.15 (0.50-2.68)	
**Chemotherapy**		*P* = .32		*P* = .97
No	Reference		Reference	
Yes	1.45 (0.70-2.99)		1.02 (0.36-2.88)	
**Trastuzumab treatment**		*P* = .61		*P* = .44
No	Reference		Reference	
Yes	1.38 (0.40-4.73)		2.54 (0.24-27.50)	
**Type**		*P* = .73		*P* = .67
Carcinoma of no special type	Reference		Reference	
Lobular carcinoma	0.86 (0.34-2.17)	*P* = .75	0.81 (0.29-2.27)	*P* = .69
Other types	0.84 (0.19-3.82)	*P* = .83	1.00 (0.19-5.36)	*P* = 1.00
Mixed/unknown	2.39 (0.46-12.36)	*P* = .30	2.88 (0.46-17.86)	*P* = .26
**Tumour size**		*P* = 1.00		*P* = 1.00
T1 (⩽20 mm)	Reference		Reference	
T2 (21-50 mm)	0.98 (0.50-1.94)	*P* = .96	0.95 (0.43-2.09)	*P* = .90
T3 (>50 mm)	0.72 (0.16-3.29)	*P* = .68	1.12 (0.21-5.91)	*P* = .89
T4	0 (0-)	*P* = 1.00	0 (0-)	*P* = 1.00
No data	0 (0-)	*P* = 1.00	0 (0-)	*P* = 1.00
**Lymph nodes**		*P* = .12		*P* = .21
N0/submicro/micro	Reference		Reference	
N1-3	0.42 (0.17-1.04)	*P* = .06	0.38 (0.13-1.12)	*P* = .08
No data	1.54 (0.41-5.76)	*P* = .52	1.02 (0.21-4.88)	*P* = .98
**Grade**		*P* = .69		*P* = .39
Grade 1	Reference		Reference	
Grade 2	1.39 (0.55-3.51)	*P* = .48	1.97 (0.69-5.62)	*P* = .20
Grade 3	0.95 (0.27-3.33)	*P* = .94	1.25 (0.28-5.46)	*P* = .77
No data	3.06 (0.27-34.19)	*P* = .36	6.40 (0.37-111.76)	*P* = .20
**HER2**				
Negative	Reference	*P* = .95	Reference	*P* = .37
Positive	1.03 (0.38-2.78)		0.43 (0.07-2.79)	
**Endocrine therapy at start**		*P* = .86		*P* = 1.00
Tamoxifen	Reference		Reference	
AI	0.84 (0.44-1.58)	*P* = .58	1.04 (0.41-2.60)	*P* = .93
Other^a^	0 (0-)	*P* = 1.00	0 (0-)	*P* = 1.00
**Cause of first change of ET**		*P* **=** **.02**		*P* **=** **.03**
No change	Reference		Reference	
Side effects	2.41 (1.17-4.96)	*P* **=** **.02**	3.13 (1.30-7.50)	*P* **=** **.01**
Clinical causes	0.52 (0.07-4.16)	*P* = .54	0.70 (0.07-6.65)	*P* = .76
Planned change	0.48 (0.16-1.45)	*P* = .20	0.64 (0.17-2.35)	*P* = .50
**Follow-up**		*P* **=** **.004**		*P* **=** **.02**
Follow-up B	Reference		Reference	
Follow-up A	2.56 (1.34-4.89)		4.48 (1.33-15.02)	

Footnote. For changed endocrine therapy ‘clinical causes’ include recurrence, oophorectomy and potentiation of therapy. Binary logistic regression analysis was performed on factors correlating with non-adherence. AOR = adjusted odds ratio; CI = confidence interval; HRT = hormone replacement therapy.

a Tamoxifen + salpingo-oophorectomy.

Being on systemic hormone replacement therapy (HRT) tended to be negatively associated with adherence in univariate regression analysis but was not significant in multivariate analysis (Table 3). Adherence to ET did not depend on characteristics of the tumour such as tumour type, size, grade, HER2 status, nor lymph node status or adjuvant treatments including radiotherapy, chemotherapy and trastuzumab therapy. Neither was age or first choice of ET predictive to adherence (Table 3). The most common reported causes related to interruption of ET were arthralgia/myalgia (23.7%), sweating/hot flushes (21.1%), depression (18.4%) and tiredness/lack of energy (18.4%; Table 4).

**Table 4.** Side effects of 38 patients terminating in advance endocrine therapy.

Side effects in 38 patients who interrupted in advance	Number of patients (%)
Arthralgia/Myalgia	9 (23.7)
Sweating/Hot flushes	8 (21.1)
Depression	7 (18.4)
Tiredness/Lack of energy	7 (18.4)
Gastrointestinal symptoms	4 (10.5)
Skin symptoms/Rashes	4 (10.5)
Oedema	3 (7.9)
Paraesthesia	2 (5.3)
Weight gain	2 (5.3)
Insomnia	1 (2.6)
Alopecia	1 (2.6)
Vertigo	1 (2.6)
Urinary tract infection	1 (2.6)
Unspecified side effects	9 (23.7)

Patients under follow-up routine A and B constituted 33.7% (119) and 66.3% (234) of the cohort, respectively. Among non-adherent patients under follow-up routine A, 52.2% of the non-adherent patients had a discussion with their physician and a mutual understanding in the decision to stop ET, whereas 47.8% stopped treatment without the recommendation or knowledge of their physician. The corresponding figures in patients under follow-up routine B were 35.0% and 65.0%, respectively (*P* = .26 between follow-up routines A and B). For patients where physicians were involved in the decision to stop ET compared with other patients, attendance at Varberg Hospital was more common (78.9% vs 49.4%, respectively, *P* = .01), follow-up routine A was more common (63.2% vs 32.0%, respectively, *P* = .007), patients had more often a previous cancer (21.1% vs 6.0%, respectively, *P* = .03), more patients tended to be prescribed tamoxifen as first ET (68.4% vs 41.0%, respectively, *P* = .06) and more patients changed their ET (*P* = .03, Table 5). In patients under follow-up routine A, 18.5% stopped ET due to side effects and 14.5% due to progression or death. The corresponding figures in patients under follow-up routine B were 6.8% and 6.8%, respectively (*P* < .0001 between follow-up routines A and B).

**Table 5.** Factors correlating with recommendation by physician to stop endocrine therapy.

	Patients recommended to stop ET by their physician (%)	All other patients (%)
**Hospital**		*P* **=** **.01**
Halmstad	4 (21.1)	169 (50.6)
Varberg	15 (78.9)	165 (49.4)
**Age groups**		*P* = .76
31-40	0 (0)	6 (1.8)
41-50	1 (5.3)	38 (11.4)
51-60	4 (21.1)	72 (21.6)
61-70	7 (36.8)	113 (33.8)
71-80	4 (21.1)	80 (24.0)
81-90	3 (15.8)	25 (7.5)
**Hormone replacement therapy at diagnosis**		*P* = .09
No	3 (15.8)	117 (35.0)
Yes-systemic	4 (21.1)	24 (7.2)
Yes-local	1 (5.3)	22 (6.6)
No data	11 (57.9)	171 (51.2)
**Previous cancer**		*P* **=** **.03**
No	15 (78.9)	314 (94.0)
Yes	4 (21.1)	20 (6.0)
**Chemotherapy**		*P* = .09
Yes	16 (84.2)	233 (66.8)
No	3 (15.8)	111 (33.2)
**Radiotherapy**		*P* = .25
Yes	12 (63.2)	243 (72.8)
No	7 (36.8)	91 (27.2)
**Trastuzumab treatment**		*P* = .47
Yes	1 (5.3)	31 (9.3)
No	18 (94.7)	303 (90.7)
**Type**		*P* = .85
Carcinoma of no special type	14 (73.7)	256 (76.6)
Lobular carcinoma	4 (21.1)	52 (15.6)
Other types	1 (5.3)	18 (5.4)
Mixed/unknown	0 (0)	8 (2.4)
**Tumour size**		*P* = .79
T1 (⩽20 mm)	12 (63.2)	193 (57.8)
T2 (21–50 mm)	7 (36.8)	113 (33.8)
T3 (>50 mm)	0 (0)	21 (6.3)
T4	0 (0)	5 (1.5)
No data	0 (0)	2 (0.6)
**Lymph nodes** ^a^		*P* = .25
N0/submicro/micro	14 (73.7)	230 (68.9)
N1-3	3 (15.8)	91 (27.2)
No data	2 (10.5)	13 (3.9)
**Grade**		*P* = .22
Grade 1	4 (21.1)	57 (17.1)
Grade 2	13 (68.4)	222 (66.5)
Grade 3	1 (5.3)	52 (15.6)
No data	1 (5.3)	3 (0.9)
**HER2**		*P* = .40
Negative	16 (84.2)	295 (88.3)
Positive	3 (15.8)	39 (11.7)
**Endocrine therapy at start**		*P* = .06
Tamoxifen	13 (68.4)	137 (41.0)
AI	6 (31.6)	196 (58.7)
Fulvestrant	0 (0)	1 (0.3)
**Changed endocrine therapy**		*P* **=** **.03**
Yes-side effects	8 (42.1)	56 (16.8)
Yes-recurrence	1 (5.3)	15 (4.5)
Yes-planned change	1 (5.3)	68 (20.4)
No	9 (47.4)	195 (58.4)
**Follow-up**		*P* **=** **.007**
Follow-up B	12 (63.2)	107 (32.0)
Follow-up A	7 (36.8)	227 (68.0)

Footnote. *P* values were determined with the 2-sided χ^2^-test or the Fisher exact test.

a Not included are patients not lymph node operated or where sentinel node was not found.

## Discussion

In this study, we wanted to explore the natural history of adherence to ET. We show that adherence to ET in BC patients is high in Halland Region. Our study indicates that patients with less nurse-initiated contact may have better adherence to ET compared with patients with more nurse-initiated contact. No differences were observed in adherence and in survival between patients initiating tamoxifen and AI in the adjuvant setting. However, change of ET from AI to tamoxifen was more common than from tamoxifen to AI.

Previous HRT tended to be associated with non-adherence to ET as shown in previous studies.^9,20^ This can be explained by that women on HRT tend to develop intolerable symptoms due to low oestrogen. More information and support during ET are needed to elevate compliance in this group. All patients in our studied cohort were given information orally and in written form of the pathological and clinical data of the tumour. In contrast to previous studies,^9^ characteristics of the tumour such as tumour size, grade and lymph node metastasis as well as adjuvant treatment were not associated with adherence. Our study may, however, have been underpowered to assess these correlations. Studies show that young women treated for early BC have worse adherence to ET compared with older women.^21-24^ The number of women in our cohort younger than 40 years constituted only 1.7%, and therefore, adherence in this age group was difficult to assess. Exposure to adjuvant chemotherapy has in some studies been identified as a negative predictor of adherence in early BC in women younger than 40 years of age.^25^ Other studies have instead shown that it is a positive predictor of adherence.^9^ However, in line with our study, several studies have not been able to show an association between adjuvant chemotherapy and adherence.^22-24^

Patients on AI experienced more side effects than patients on tamoxifen. Experiencing side effects of ET was also an independent factor correlating with worse adherence. It was more common to change from AI to tamoxifen than the opposite direction. As earlier studies have shown,^20^ changing ET due to side effects was correlated with poorer adherence. This appears logical as patients normally are prescribed another type of ET if intolerable side effects appear before interrupting treatment. However, whether patients started ET with tamoxifen or AI did not seem to be associated with adherence to ET.

We report a relatively high persistence to ET in the adjuvant setting of BC where medication persistence for 4 years and more was 75.7% for patients initiating AI and 72.0% for patients initiating tamoxifen. To note, compliance figures vary between studies, ie, compliance in the adjuvant setting of BC has been reported to be 69% in Regions Stockholm-Gotland and Region Uppsala-Örebro, whereas compliance was 92% in Region Jönköping in Sweden.^26,27^ Variations of compliance may not only be due to any differences in patient cohorts but also how compliance is defined. Moreover, treatment traditions at different clinics may also contribute to compliance.

Earlier studies suggest that high availability and information about benefits of ET and potential side effects are of importance to compliance,^28^ but exactly how follow-up should be arranged to result in best compliance is not known. Breast cancer patients adherent to ET are more likely to contact their nurse when they experience side effects to ET.^16^ This implies that availability of health care staff is important to improve adherence. In our study, all patients, regardless of follow-up routine, were given contact details to a specialized nurse and were informed to call in case of side effects or if they had any questions about their ET. This available support and consult may have contributed to the high adherence observed in the cohort. To our surprise, a more active monitoring, where the nurse contacted the patient annually, more often led to a poorer adherence to ET compared with a more patient-controlled follow-up. However, the percentage in decisions where physicians were involved in ending ET and the percentage in decisions where patients decided independently to end ET were the same in patients under follow-up routines A and B. Therefore, we cannot conclude that lower adherence in patients under follow-up routine A solely depended on physicians and nurses driving patients to end ET. The contact may, however, have triggered the patient independently to end ET. When contacted by their nurse, patients had the opportunity to discuss their ET and any side effects, where quality of life was weighted against potential benefits of continued ET based on patient and tumour factors.

The strength of our study is that we were able to track almost all patients during follow-up and analysed how changes in ET occur over time. The follow-up time varied depending on date of surgery and if the patient underwent chemotherapy before starting ET. Most patients in routine A were included in 2015 (69.7%), whereas most patients in routine B (60.7%) were included in 2016. This means that participants included in 2016 had less time to become non-adherent than participants included in 2015. However, there was no difference in follow-up time between the 2 assessed follow-up routines where patients from 2015 and 2016 were combined. A limitation of the study is that the sample size was fixed to all patients treated for breast lump in our region between 2015 and 2016, and no power analysis was therefore performed. To assess adherence, we analysed whether patients had asked for new prescriptions of ET, however, we did not record whether the prescribed ET was collected from the pharmacy, and this is a bias in our study. Breast cancer–specific survival could also have been over-estimated in our study as cases were retrieved only from the medical records of the patients. However, as migration was low in our cohort, we are convinced that our estimate of survival was close to accurate. The study was conducted in two hospitals and a small number of physicians were involved in treatment decisions. Therefore, we cannot rule out that treatment traditions in physicians and nurses working at the two clinics may have affected the observed adherence differences between follow-up routines A and B. To note, how tamoxifen vs AI was used and the involvement of physicians in the decision to stop ET were different between the two hospitals assessed. Compliance differences in the regions of Sweden have also been observed in previous studies.^9^

## Conclusions

Our study shows that adherence to ET is high in Region Halland and that follow-up routines are of importance for adherence. Treatment traditions at clinics may affect adherence to ET. Randomized controlled studies assessing the best follow-up routine in the adjuvant of BC are therefore warranted.

## Declarations

**Ethics approval and consent to participate:** The Regional Ethical Review Board in Gothenburg, Sweden, approved the present study (Dnr 2021-02898). Informed consent from participants was waived in view of the retrospective design of the study and the minimal risk to the subjects of participating.

**Consent for publication:** All data are reported in an aggregate manner, ie, no individual data of persons can be identified.

**Availability of data and materials:** Additional data can be available upon request to the Institute of Clinical Sciences, Sahlgrenska Academy at the University of Gothenburg.
